# Use of preoperative embolization prior to Transplant nephrectomy

**DOI:** 10.1590/S1677-5538.IBJU.2015.0052

**Published:** 2016

**Authors:** Carrie Yeast, Julie M. Riley, Joshua Holyoak, Gilbert Ross, Stephen Weinstein, Mark Wakefield

**Affiliations:** 1 Department of Urology, University of Missouri, Missouri, USA;; 2 UNM - Surgery Albuquerque, New Mexico, USA

**Keywords:** Transplants, Nephrectomy, Embolization, Therapeutic

## Abstract

**Introduction:**

After a failed transplant, management of a non-functional graft with pain or recurrent infections can be challenging. Transplant nephrectomy (TN) can be a morbid procedure with the potential for significant blood loss. Embolization of the renal artery alone has been proposed as a method of reducing complications from an in vivo failed kidney transplant. While this does yield less morbidity, it may not address an infected graft or refractory hematuria or rejection. We elected to begin preoperative embolization to assess if this would help decrease the blood loss and transfusion rate associated with TN.

**Materials and Methods:**

We performed a retrospective analysis of all patients who underwent non-emergent TN at our institution. Patients who had functioning grafts that later failed were included in analysis. TN was performed for recurrent infections, pain or hematuria. We evaluated for blood loss (EBL) during TN, transfusion rate and length of hospital stay.

**Results:**

A total of 16 patients were identified. Nine had preoperative embolization or no blood flow to the graft prior to TN. The remaining 7 did not have preoperative embolization. The shortest time from transplant to TN was 8 months and the longest 18 years with an average of 6.3 years. Average EBL for the embolized patients (ETN) was 143.9cc compared to 621.4cc in the non-embolized (NETN) group (p=0.041). Average number of units of blood transfused was 0.44 in the ETN with only 3/9 patients requiring transfusion. The NETN patients had average of 1.29 units transfused with 5/7 requiring transfusion. The length of stay was longer for the ETN (5.4 days) compared to 3.9 in the NETN. No intraoperative complications were seen in either group and only one patient had a postoperative ileus in the NETN.

**Conclusion:**

Embolization prior to TN significantly decreases the EBL but does not significantly decrease transfusion rate. However, patients do require a significantly longer hospitalization with embolization due to the time needed for embolization. Larger studies are needed to determine if embolization before transplant nephrectomy reduces the transfusion rates and overall complications.

## INTRODUCTION

Although there have been vast improvements in surgical and immunosuppressive techniques in kidney transplant, transplant failure is still a significant obstacle. Rates of graft survival beyond 5 years are largely unchanged (1). While rejection is the most common cause of transplant failure, other causes include infection and recurrence of previous kidney disease (2). When the renal graft fails, several concerns can develop including pain, hematuria, and/or infections. Options for these patients traditionally have only been transplant nephrectomy or supportive care. Recently, embolization alone has shown to be beneficial in chronic pain and hematuria. However, it is not always successful in improving these symptoms. Earlier studies have shown that embolization prior to nephrectomy can eliminate some of the morbidity associated with transplant nephrectomy (1).

Transplant nephrectomy can be a morbid procedure with extensive blood loss and potential for intra and post-operative complications. The overall reported morbidity of transplant nephrectomy ranges from 4.3 to 84.4% and mortality rates have been quoted to be between 1.2 and 38% (1). Vascular complications associated with transplant nephrectomy include hemorrhage, pseudoaneurysm, or death (3).

The presence of a failed allograft in vivo can be associated with pain, chronic infection, and even sepsis. A retained failed allograft in vivo leads to elevated ESR and CRP. Chronically, this can lead to erythropoietin resistance, decreased albumin, and malnutrition (4). Some studies have shown failed transplants that remain in vivo continue to produce anti-HLA immunoglobulin, maintaining the inflammatory response. There have been conflicting studies as to whether transplantectomy affects PRA levels or changes re-transplant graft survival rates (5). The presence of a failed allograft when a patient must return to dialysis is associated with anemia and hypoalbuminemia, which increases the risk of poor outcomes (4).

Recent studies determined that nephrectomy of a failed allograft does not seem to significantly influence the survival of a subsequent graft (6). However, in a recent large study of transplant patients that returned to dialysis after failed kidney transplant, receipt of allograft nephrectomy was associated with a 32% lower adjusted relative risk for all causes of death. (7). Therefore, it seems transplant nephrectomy in a patient who is a surgical candidate may be preferred. However, attempts to reduce the morbidity and mortality of the procedure are still being investigated.

We proposed that performing preoperative embolization prior to transplant nephrectomy may reduce the morbidity associated with this procedure.

## MATERIALS AND METHODS

Data was collected retrospectively on consecutive non-emergent transplant nephrectomies performed at our institution between the years of 2001 and 2013.

Beginning in 2006, evaluation of blood flow to the failed graft was assessed using Doppler ultrasound. This was considered Group-1 regardless of flow status. If flow was identified, these patients went on to have embolization of the graft followed by transplant nephrectomy. If no flow was identified, patients proceeded onto transplant nephrectomy alone. Prior to 2006, no evaluation of blood flow was made and no patients underwent preoperative embolization. This was considered Group-2.

The embolization was performed by interventional radiology. This was done by a standardized technique with access via the right femoral artery. Gelfoam™ slurry was used in most cases to perform the embolization. One case each utilized Embospheres® microspheres or Tornado™ embolization coils. This was done at the preference of the interventional radiologist. Transplant nephrectomy was performed between 1 and 17 days after embolization for all but one patient. Four of the patients in Group-1 remained in the hospital between embolization and nephrectomy. One patient had surgery thirteen months after embolization due to persistent pain at the graft site after initial improvement. Transplant nephrectomy was performed through a modified Gibson incision utilizing the incision performed at the time of transplantation. All surgeries in both groups were performed extraperitoneally, using a subcapsular technique. Clamp hilar control was obtained as fast as safely possible utilizing vascular clamps. The kidney was removed and the vessels were oversewn. After surgery, all patients regardless of preoperative renal blood flow status discontinued immunosuppressive agents.

A retrospective analysis of the data was performed. Endpoints evaluated included estimated blood loss (EBL), blood transfusion rates, length of hospital stay and peri-and postoperative complications.

## RESULTS

Sixteen consecutive non-emergent transplant nephrectomies performed at our institution between the years of 2001 and 2013 were included in the analysis. Nephrectomy for patients in the immediate post-transplant period for acute thrombosis, bleeding, or infectious complications were not analyzed in this setting. Nine patients underwent preoperative embolization or were determined preoperatively to have no blood flow on Doppler ultrasound and were defined as Group-1. Within Group-1, three had no blood flow to the renal artery on preoperative evaluation and six showed flow and subsequently had preoperative renal embolization. Group-2 contained seven patients who did not receive embolization or evaluation of flow prior to nephrectomy. Transplant nephrectomy was performed for recurrent infections in seven, pain in four, hematuria in one, a combination of pain and hematuria in two, persistent anemia in one and disease recurrence in one. Table-1 lists the breakdown of patients in each group. Group-1 had significantly longer time from transplant until nephrectomy (p=0.0023). All transplantectomies in both groups were performed at least 6 months after initial graft placement. The age of the two groups ranged from 22-69 and there was no significant difference between them (p=0.763).

**Table 1:** Patient Demographics.

Patient	Age at Nephrectomy	Cause of ESRD	Indication for Nx	Time to nephrectomy (years)	Presence of RBF
Group-1					
1	47	HTN	Recurrent UTI	9	Yes
2	58	PCKD	Gross hematuria	18	Yes
3	32	FSGS	Repeat Treatment	12	No
4	20	MPGN	Recurrent UTI	5	No
5	69	HTN	Recurrent UTI	8	No
6	45	HTN	Hematuria, pain	8	Yes
7	40	DM	Pain	8	Yes
8	22	PCKD	Infection	3	Yes
9	51	DM	Pain	8	Yes
Average	42.6			8.78	

Group-2					

1	61	DM	Pain, hematuria	6	Not assessed
2	26	HTN	Pyelonephritis	2	Not assessed
3	67	FSGS	Infection	0.7	Not assessed
4	35	HTN	Pain	3	Not assessed
5	38	PCKD	Pain	1.5	Not assessed
6	38	HTN	Anemia	2	Not assessed
7	44	HTN	Unknown	Unknown	Not assessed
Average	44.1			2.53	
P value	0.76			0.0051	

**ESRD =** End Stage Renal Disease; **Nx=** Nephrectomy; **DM =** Diabetes Mellitus; **HTN =** Hypertension; **PCKD =** Polycystic Kidney Disease; **FSGS =** Focal Segmental Glomerulosclerosis; **UTI =** Urinary Tract Infection; **MPGN =** Membrano-proliferative glomerulonephritis.

Group-1 had an average estimated blood loss of 143.89 ccs (range 20-475ccs) and Group-2 lost an average of 621.4ccs (range 50-1500ccs). This difference was significant with a p value of 0.047. However, the number of blood units given was not statistically different with an average of 0.5 units (range 0-2) transfused in Group-1 and 1.29 units (range 0-3) in Group-2 (p-value=0.214). Three of the nine patients in Group-1 required transfusion while five out of the seven in Group-2 required transfusion.

The average intraoperative time in Group-1 was just over 132 minutes. Unfortunately, adequate documentation of intraoperative time was not available for most patients in Group-2. Group-1 had a significantly longer hospitalization at an average of 5.5 days (range 4-7 days). In comparison, Group-2 stayed on average 3.86 days (Range 2-5 days). There was a significant difference with a p value of 0.013 probably due to the fact that several patients received embolization prior to nephrectomy but during the same hospital stay.

No peri-or postoperative complications were seen in Group-1. Group-2 had one patient that experienced a postoperative ileus which was resolved by postoperative day three and required the patient to stay five total days postoperatively.

## DISCUSSION

Approximately 6-16% of transplanted kidneys eventually required explantation (1). This difficult surgery has several known complications, including bleeding, abscesses, wound infection, and vascular injury. This study proves that embolization before nephrectomy reduces blood loss but does not prove to decrease overall transfusion rates.

The estimated blood loss of the embolized patients was significant less than the non-embolized group. However, overall transfusion rates were not significantly different. All patients in the embolized group underwent nephrectomy more recently than the non-embolized group. While the basic surgical technique did not significantly change, improvements in surgical methods, transfusion practices, and particular surgeon’s preferences may have changed somewhat between the earlier years when the non-embolized nephrectomies occurred and the later years when embolization began. Most transplant centers report half the use of blood products compared to ten years ago (8). This may have some effect on when blood was given to the surgical patients in this study.

While transfusion rates were not significantly different, estimated blood loss is an important factor in overall surgical morbidity and is still an important consideration in choosing surgical techniques as blood loss is associated with increased morbidity, mortality, and hospital stay (8). Patients undergoing transplant nephrectomy are often anemic or have other comorbidities which may make total blood loss more significant even when it does not affect overall transfusion rate. Kidney disease is a major adverse prognostic factor for cardiovascular events (9). Thus, operative blood loss, which creates such complications as hemodilution, hypothermia, clotting factor consumption and acidosis can create situations that are especially precarious in this patient population (8).

**Table 2:** Surgical and Post-Operative Characteristics of Group 1 (Embolization) and Group 2 (No Embolization)

Variable	Embolization	No Embolization	P Value
Estimated Blood Loss (cc)	143.89	621.43	**0.0417**
Units of Blood Transfused	0.44	1.29	0.114
Transfusion Rate (percentage)	0.33	0.714	0.149
Total Hospital Length of Stay (days)	5.44	3.86	**0.0112**
Intraoperative Complications	0	1	0.3559

Embolization with nephrectomy was found in this study to lead to longer hospital stays. This can create a new set of concerns such as increased financial burdens and comorbidities such as hospital acquired infections that are associated with longer hospital visits. However, length of stay in the embolized group can partially be accounted for due to the fact that two procedures were performed. Despite longer stays, lack of intra and postoperative bleeding lowers the risk for cardiovascular and hemodynamic complications (8). Surgical approaches allowing embolization and nephrectomy as one procedure under general anesthesia should be investigated to determine if hospital stays are still longer than nephrectomy alone.

Doppler ultrasound looking for transplant renal blood flow was not routinely performed before nephrectomy until 2006. This practice began as a new surgeon elected to do ultrasounds prior to nephrectomy as standard technique. Therefore, it is reasonable to believe that some of the patients in Group-2 also had no blood flow, placing them in the incorrect study group and possibly altering study results.

Further studies with larger groups are necessary to evaluate whether embolization affects overall complications with transplant nephrectomy. Larger groups have been reviewed in transplant nephrectomy alone without regard to embolization. The paper, “Review of a Transplantectomy Series” published in Transplantation Proceedings in 2015 reviewed 70 transplantectomies and found serious complications (Clavien>III) in 21% of all cases (10). However, this study did not address prior embolization or lack of blood flow to the kidney before surgery.

Similar studies to ours have used comparative numbers to this study. The paper, “Intraoperative Coil Embolization Reduces Transplant Nephrectomy Transfusion Requirement” published in Aug 2007 in Vascular and Endovascular Surgery had data on 13 consecutive patients with similar results found in this study (1). Another study published in Nefrologia in 2005 addressed embolization alone versus nephrectomy and had a study population of seven (11). Thus, although our study was limited by its small numbers, the rarity of this surgery makes this one of the larger study populations examined. A multi-center study may be necessary to more accurately look at peri and post-operative complications.

This study was also limited by its retrospective nature. However, prospective studies would again be limited by the infrequent occurrence of this surgery.

## CONCLUSIONS

Embolization of the renal transplant prior to nephrectomy is beneficial given that there is less blood loss. However, patients may require a longer hospitalization when embolization is performed as a separate procedure prior to nephrectomy. Larger prospective studies are needed to validate our results as well as to determine if embolization before transplant nephrectomy reduces the transfusion rates and overall complications.
